# In Vitro Assessment of Fluconazole and Cyclosporine A Antifungal Activities: A Promising Drug Combination Against Different *Candida* Species

**DOI:** 10.3390/jof11020133

**Published:** 2025-02-10

**Authors:** Juan Daniel Carton, Iñigo de-la-Fuente, Elena Sevillano, Nerea Jauregizar, Guillermo Quindós, Elena Eraso, Andrea Guridi

**Affiliations:** 1 Department of Immunology, Microbiology and Parasitology, Faculty of Medicine and Nursing, University of the Basque Country UPV/EHU, 48940 Leioa, Spain; juandaniel7c@gmail.com (J.D.C.); idelafuente05@hotmail.com (I.d.-l.-F.); guillermo.quindos@ehu.eus (G.Q.); elena.eraso@ehu.eus (E.E.); andrea.guridi@ehu.eus (A.G.); 2 Department of Pharmacology, Faculty of Medicine and Nursing, University of the Basque Country UPV/EHU, 48940 Leioa, Spain; nerea.jauregizar@ehu.eus; 3 Biobizkaia, Basque Health Research, 48903 Barakaldo, Spain

**Keywords:** *Candida*, cyclosporine A, fluconazole, drug combination, synergism, time–kill, checkerboard technique

## Abstract

Invasive candidiasis is a common fungal infection associated with multiple risk factors, such as cancer, neutropenia, corticosteroid therapy, catheterization, and the use of broad-spectrum antibiotic treatment. *Candida albicans* is the predominant causative agent, although other *Candida* species have been emerging in the last years, together with a rise in a number of strains resistant to the currently available antifungal drugs, which poses a challenge when treating these infections. Drug repurposing and drug combinations are promising strategies for the treatment of invasive mycoses. In this study, we evaluated the effect of the combination of fluconazole (FLZ) and cyclosporine A (CsA) against 39 clinical isolates and reference strains of *Candida*. Two methods, the Loewe additivity model and Bliss independence model, were used to assess the antifungal activity of the drug combination according to CLSI and EUCAST guidelines. The results demonstrated a synergistic effect between fluconazole (FLZ) and cyclosporine A (CsA) against 15–17 *Candida* isolates, depending on the evaluation model used, including FLZ-resistant strains of *C. albicans*, *C. glabrata*, *C. parapsilosis*, and *C. tropicalis*. Notably, the combination significantly reduced the minimum inhibitory concentration (MIC) of FLZ in a substantial number of isolates, including those with resistance to FLZ. Additionally, time–kill curve studies confirmed the synergistic interaction, further validating the potential of this combination as an alternative therapeutic strategy for candidiasis treatment. These findings emphasize the importance of investigating innovative drug combinations to address the challenges posed by antifungal resistance and improve treatment options for invasive fungal infections.

## 1. Introduction

The incidence of invasive candidiasis continues to rise, largely due to an upsurge in certain high-risk medical conditions and treatments. Factors such as cancer, prolonged neutropenia, widespread catheterization, and extensive use of corticosteroids and/or broad-spectrum antibiotics have all contributed to growing patient populations in which *Candida* infections can thrive [1,2].

Invasive candidiasis is related to a high mortality rate, affecting up to 40% of critically ill patients in intensive care units (ICU). *Candida albicans* remains the most prevalent etiological agent, although there is an ongoing epidemiological shift marked by a notable rise in infections caused by other species of *Candida* [3]. The global health threat of invasive fungal diseases is related to a rapid emergence of resistant isolates and/or species of *Candida*. In fact, there are six *Candida* species in the fungal priority pathogens list released by the World Health Organization (WHO) [4]. *C. albicans* and *Candida auris* (currently *Candidozyma auris*) are classified as critical priority pathogens in this list; *Candida glabrata* (currently *Nakaseomyces glabratus*), *Candida parapsilosis,* and *Candida tropicalis* are classified in the high priority group, and finally, *Candida krusei* (currently *Pichia kudriavzeveii*) is classified in the medium priority group [4]. These species often show varying levels of resistance to standard antifungal drugs, complicating clinical management and contributing to the overall rise in antifungal resistance and therapeutical failures [5,6].

Therapeutic options for treating mycoses are more limited compared to those available for bacterial infections. The azoles represent the largest class of antifungal drugs and are very effective against a broad spectrum of candidiasis, including those affecting the skin or mucous membranes (vaginal and cutaneous candidiasis) as well as more severe invasive infections. Fluconazole (FLZ), a member of this class, inhibits a key enzyme in ergosterol biosynthesis, lanosterol 14α-demethylase (CYP51A1), which is essential for fungal cell membrane integrity [7]. It is widely used due to its high efficacy, low toxicity, minimal drug interactions, and excellent oral bioavailability. However, the emergence of azole-resistant *Candida* strains and species with reduced susceptibility to FLZ, such as the multidrug-resistant *C. auris*, presents an increasing therapeutic challenge [4,8].

To overcome this problem, several strategies have been proposed, including the search for new antifungal agents, repurposing existing non-antifungal drugs, improving formulations of current treatments, or using combinations of antifungal and non-antifungal agents. One promising approach highlighted by researchers is to enhance the effectiveness of FLZ by combining it with other agents, potentially providing a more effective treatment for fungal infections [9].

Calcineurin is a calcium/calmodulin-dependent protein phosphatase that regulates fungal physiology. It is involved in cation homeostasis, morphogenesis, cell wall biosynthesis, antifungal drug resistance, and virulence [10,11]. Therefore, pharmacological inhibition of fungal calcineurin mediated by cyclosporine A (CsA) is a strategy used to inhibit calcineurin activity [12,13,14]. CsA was first isolated from the fungus *Tolypocladium inflatum* and acts as an immunosuppressant, inhibiting the activity of calcineurin through its interaction with the immunophilins, specifically cyclophilin A, in immunocompetent lymphocytes. Previous studies showed that FLZ combined with CsA exhibited a potent fungicidal synergism in vivo and in vitro against some *C. parapsilosis* and *C. albicans* strains [12,13]. The aims of this study were to evaluate the in vitro interaction between FLZ and CsA against clinical isolates and reference strains of different *Candida* species, and to compare two methods to assess the antifungal activity of drug combinations—the Loewe additivity model (calculating the fractional inhibitory concentration index) and Bliss independence model.

## 2. Materials and Methods

### 2.1. Fungal Strains

The study included a total of 37 clinical isolates, comprising *C. albicans* (n = 10), *C. auris* (n = 5), *C. glabrata* (n = 7), *C. parapsilosis* (n = 6), *C. krusei* (n = 3), *Candida nivariensis* (currently *Nakaseomyces nivariensis*) (n = 2), *Candida orthopsilosis* (n = 2), and *C. tropicalis* (n = 2) (additional information available in Table S1). *C. krusei* ATCC 6258 and *C. parapsilosis* ATCC 22019 were used as quality control. This sample distribution ensured the inclusion of species with varying susceptibility profiles and clinical relevance. The sample sizes used in this study were selected to provide a robust and statistically significant representation of different *Candida* species, encompassing both commonly encountered and emerging clinical isolates.

Clinical isolates were obtained from the fungal strain culture collection of the Medical Mycology Laboratory from the University of the Basque Country (UPV/EHU, Bilbao, Spain), and *C. auris* isolates were obtained from the Microbiology Service of La Fe University and Polytechnic Hospital (Valencia, Spain). Clinical isolates and collection strains were stored in distilled sterile water and subcultured on Sabouraud dextrose agar (SDA).

### 2.2. Drugs Tested

The antifungal drug used in this study was FLZ (Sigma-Aldrich, Madrid, Spain), with a final concentration ranging from 0.25 to 64 µg/mL, prepared according to the manufacturer’s recommendations. Additionally, CsA (Sigma-Aldrich, Madrid, Spain) was used in concentrations ranging from 0.25 to 128 µg/mL.

### 2.3. Antifungal Susceptibility Studies

The minimal inhibitory concentration (MIC) was determined using the broth microdilution method described in the CLSI M27-A3, M27-S4, and EUCAST EDef 7.3.1 guidelines [15,16]. MICs were defined as the lowest drug concentrations that resulted in a 50% reduction of visible fungal growth after 24 h of incubation. For the EUCAST method, RPMI 1640 supplemented with L-arginine and 2% glucose and buffered with 0.165 M morpholinopropanesulfonic acid (MOPS) was used as the culture medium. In contrast, the CLSI method used RPMI 1640 supplemented with L-arginine but only 0.2% glucose. After adjusting the pH to 7.0 ± 0.1, the medium was sterilized by filtration and stored at 4 °C. To perform the assays, a stock solution of the compounds was prepared in dimethyl sulfoxide (DMSO). The range of drug concentrations varied from 0.25 to 64 μg/mL for FLZ and 0.25 to 128 μg/mL for CsA.

### 2.4. Checkerboard Assay

The in vitro susceptibility of the clinical isolates was tested against the combination of FLZ and CsA using the checkerboard method to determine the minimum inhibitory concentration (MIC). The tests followed the protocols described in CLSI documents M27-A3 and M27-S4 and EUCAST document EDef 7.3.1, modified for drug combinations, using flat and U-bottom 96-well microtiter plates, respectively.

CsA was dispensed into columns 2–11 of the plates, with concentrations ranging from 0.25 to 128 µg/mL. FLZ was added in rows A–G, at concentrations ranging from 0.25 to 64 µg/mL. Wells in column 12 served as growth controls, with 100 µL of RPMI supplemented with 2% or 0.2% glucose (according to EUCAST or CLSI protocols, respectively) and 2% DMSO. Wells H1 and H12 were used as sterility controls.

*Candida* isolates, previously incubated overnight at 37 °C, were suspended in distilled water to achieve an inoculum of 0.5–2.5 × 10^3^ CFU/mL in the CLSI method and 0.5–2.5 × 10^5^ CFU/mL in the EUCAST method. Subsequently, 100 µL of these suspensions were inoculated into each well of the microtiter plates, excluding H1 and H12. The plates were incubated at 37 °C for 48 h and the absorbance of each well was measured using an Infinite F50 spectrophotometer (Tecan, Männedorf, Switzerland) at a wavelength of 450 nm. Each experiment was performed in triplicate and carried out in three independent assays.

### 2.5. Statistical Analysis

#### 2.5.1. FICI Model

The effect of the combination was interpreted according to Loewe’s additive theory, which postulates that a drug does not interact with itself. According to this model, two drugs are considered additive if the combined effect of the drugs is equivalent to the sum of their individual effects when adjusted for potency differences. It involves a comparative analysis of the concentrations of the drugs, both individually and in combination. The fractional inhibitory concentration index (FICI) is mathematically represented by the following equation:

∑FIC = FIC_A_ + FIC_B_ = C_A_^comb^/MIC_A_^alone^ + C_B_^comb^/MIC_B_^alone^

where MIC_A_^alone^ and MIC_B_^alone^ are the MIC of drugs A and B acting alone, and C_A_^comb^ and C_B_^comb^ are concentrations of drugs A and B at the isoeffective combinations. The interpretation of the FICI was as follows: A synergistic interaction occurs when the combination of two compounds produces a significantly greater effect than the sum of their individual effects, represented by a FICI value of <0.5. An additive interaction is observed when the combined effect is approximately equal to the sum of the effects of each compound alone, with a FICI value between 0.5 and <1. An indifferent interaction is characterized by the combination having an effect similar to that of the most active compound alone, without any significant enhancement or interference, corresponding to a FICI value between 1 and <4. Finally, an antagonistic interaction occurs when the combined effect is weaker than the effect of the most active compound, indicating that one compound interferes with the action of the other; this is defined by a FICI value of ≥4 [17,18].

#### 2.5.2. Bliss Independence Model

The results were also analyzed according to the Bliss independence theory, which is a reference model used to determine the expected effect of two drugs acting independently without any interaction. The principle behind Bliss independence is that the combined effect of two drugs should be equal to the sum of their individual effects if they are acting independently. This model uses the variation of growth percentage (ΔE), which describes the interaction as the difference between the expected and observed growth percentage for each drug combination. The non-parametric method of Bliss independence is based on the Prichard model [19] and was described by the following equation:

Ii = (I_A_ + I_B_) − (I_A_ × I_B_)

where Ii is the expected percentage of inhibition of the combination of drug A and B, and I_A_ and I_B_ are the experimental percentages of inhibition of each drug alone. The resulting value of ΔE provides insight into the nature of the interaction between the two drugs. The sums of the percentages of all statistically significant synergistic (∑SYN) and antagonistic (∑ANT) interactions were calculated. When the mean difference was positive, as well as its 95% confidence interval (CI) among three replicates, it was interpreted as statistically significant synergy. When the difference, as well as its 95% CI, was negative, it was interpreted as significant antagonism. In any other case, it was concluded that there was no interaction. Interactions with <100% of statistically significant interactions were considered weak, those with 100% to 200% of statistically significant interactions were considered moderate, and those with >200% of statistically significant interactions were considered strong [20]. The parameters of this model were obtained using Combenefit software v. 2.021 (University of Cambridge, Cambridge, UK).

### 2.6. Time–Kill Curve Studies

The time–kill method was carried out on microtiter plates with RPMI 1640 as previously described [21] against six isolates: two susceptible and four resistant to FLZ. The drug concentrations were selected based on the obtained MIC values to ensure that the time–kill assay effectively evaluated the fungicidal activity of the drugs. Different antifungal concentrations were prepared for the evaluation of these studies: FLZ 8 μg/mL, CsA 2 μg/mL and the combination of FLZ with CsA (8 and 2 μg/mL, respectively). After dispensing 100 μL of the antifungal suspensions, a 100 μL of the inoculum of 1–5 × 10^5^ CFU/mL was added to each well. Plates were incubated 48 h at 37 °C without agitation. Samples for colony counts were taken at intervals of 0, 2, 4, 6, 24, and 48 h after incubation, allowing for a detailed assessment of fungal growth dynamics over time. After incubation in SDA plates at 37 °C for 24 to 48 h, the number of CFU/mL were calculated. The results were expressed as mean colony counts from triplicate experiments on three separate days. The lower limit of detectable colony counts was 5 CFU/mL.

Synergism was defined as a decrease in CFU/mL of ≥2 log_10_ compared to the most active drug, indifference as a decrease in CFU/mL < 2 log_10_ compared to the most active drug, and antagonism as an increase in CFU/mL ≥ 2 log_10_ compared to the less active drug [22].

## 3. Results

The results of the susceptibility testing of FLZ and CsA in monotherapy and in combination against 37 isolates of *Candida* are summarized in Table 1 and Table 2. The MIC of FLZ in monotherapy against all isolates ranged from 1 to >64 µg/mL, while the MIC of CsA was >128 µg/mL in all cases. Among these isolates, 22 were classified as FLZ-resistant by both methods (CLSI and EUCAST). All susceptibility testing categories were concordant for the two methods, except for two isolates, *C. glabrata* UPV 15-202 (32 µg/mL (I) and 64 µg/mL (R) for EUCAST and CLSI, respectively) and *C. parapsilosis* UPV 15-177 (8 µg/mL (R) and 4 µg/mL (SDD) for EUCAST and CLSI, respectively).

When analyzing the results obtained with the CLSI method (Table 1), a reduction in FLZ MIC was observed when combined with CsA, with MIC values decreasing from 64–>64 µg/mL to 1–4 µg/mL against nine FLZ-resistant isolates. These included the following isolates: five *C. albicans*, two *C. glabrata*, one *C. parapsilosis*, and one *C. tropicalis*. FICI analysis interpreted the effect of the combination as synergistic against 16 of the 37 isolates studied (43.24%), including 11 fluconazole-resistant isolates. An additive effect was detected against 4 (two resistant, one SDD and one S) of the 37 isolates tested (10.81%). The interpretation of the two models, Bliss and Loewe models, were consistent in all the cases in which the synergistic and additive effects were observed. The effect of the combination was classified as indifferent against the five *C. auris* studied.

Similarly, when using the EUCAST method (Table 2), the same reduction in the MIC of FLZ from 64–>64 µg/mL to 1–4 µg/mL was detected when combined with CsA against ten fluconazole-resistant isolates, which included five *C. albicans*, two *C. krusei*, one *C. glabrata*, one *C. parapsilosis*, and one *C. tropicalis*. FICI analysis revealed a synergistic effect against 15 isolates (40.5%), 10 of which were resistant to FLZ, while an additive effect was observed against 5 out of the 37 isolates tested (13.5%). The combination was synergistic against all *C. nivariensis* isolates, 71.4% of *C. glabrata* isolates, or 50% of *C. albicans* isolates. When analyzing the results through the Bliss model, they were consistent with those obtained using the FICI method except in two cases (*C. glabrata* UPV 16-006 and *C. albicans* NCPF 3153 in which the interpretation was additive and synergistic, by FICI and Loewe models, respectively). In the Bliss model, ∑SIN indicated strong synergistic interactions for 17 isolates, with high values between 921 and 3349. The only exception was *C. albicans* NCPF 3153, which showed a synergistic interaction value of 203, which, although lower, is still classified as strong.

The results obtained with EUCAST were consistent with those obtained using the CLSI method, showing a similar reduction in FLZ MIC when combined with CsA. The synergy observed in both methods was consistent, with both obtaining synergistic effects against 15 and 16 isolates and additive effects against 3 and 4 isolates when using EUCAST and CLSI, respectively. The results also revealed that both methods showed a synergistic effect against all *C. nivariensis* isolates studied. On the other hand, the combination of FLZ and CsA did not exhibit synergistic effects against any isolates of *C. auris*, *C. krusei* or *C. orthopsilosis*.

**Figure 1:**
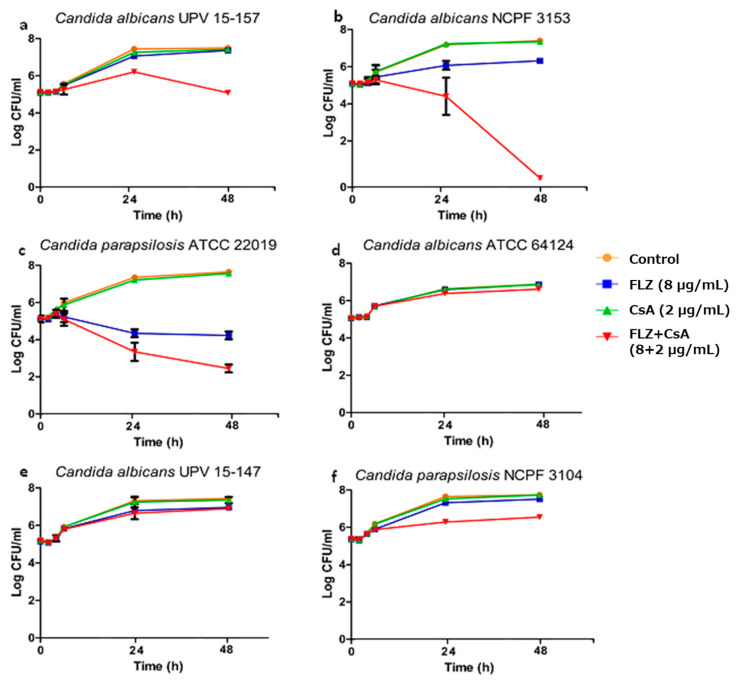
Time–kill curve plots of FLZ and CsA, alone and in combination, against two fluconazole-susceptible strains (*C. albicans* NCPF 3153 (**b**), *C. parapsilosis* ATCC 22019 (**c**)) and four fluconazole-resistant strains (*C. albicans* ATCC 64124 (**d**), *C. albicans* UPV 15-147 (**e**), *C. albicans* UPV 15-157 (**a**), and *C. parapsilosis* NCPF 3104 (**f**)).

The results obtained by the time–kill curves are shown in Figure 1, where it is represented in plots of log CFU/mL versus the duration of the assay.

This method confirmed the synergistic effect of the combination of FLZ with CsA detected by the checkerboard assay against *C. albicans* UPV 15-157 and *C. albicans* NCPF 3153. The drug combination resulted in a reduction of 2.28 log CFU/mL compared to the most effective drug in monotherapy against the fluconazole-resistant *C. albicans* UPV 15-157 isolate. Similarly, the combination reduced fungal growth 6.335 log CFU/mL against *C. albicans* NCPF 3153 after 48 h compared to FLZ in monotherapy, showing a reduction greater than 3 log CFU/mL from the initial inoculum.

As for the fluconazole-susceptible strain *C. parapsilosis* ATCC 22019, the combination showed enhanced fungistatic activity, with a reduction of 1.78 log CFU/mL relative to FLZ in monotherapy and a total decrease of 2.63 log CFU/mL from the initial inoculum.

Finally, there was no concordance between the results obtained by the checkerboard method and the time–kill curves against the strains *C. albicans* ATCC 64124, *C. albicans* UPV 15-147, and *C. parapsilosis* NCPF 3104, as while the interaction resulted additive or synergistic according to the first method, it resulted indifferent in the time–kill curves assays.

## 4. Discussion

Invasive candidiasis poses a significant medical challenge due to the increasing number of affected patients [23]. The epidemiology of this infection has shifted, with a rise in cases caused by non-*C. albicans Candida* species and the emergence of antifungal-resistant isolates, complicating treatment and patient management. To address these challenges, strategies such as drug repurposing and combinations aimed at achieving synergistic antifungal effects have gained attention [9,24]. Among these, the combination of fluconazole (FLZ) and cyclosporin A (CsA) has shown potential to enhance *Candida albicans* susceptibility to fluconazole [12,13,25]. In this study, we evaluated the effect of this combination against 37 isolates belonging to eight different *Candida* species, including the six species listed in the priority pathogens list published by the WHO [4].

Our study showed that the combination of FLZ and CsA had a synergistic effect against a significant percentage of isolates of *Candida*, including 33.3% of *C. parapsilosis*, 50% of *C. tropicalis*, 60% of *C. albicans*, 71.4% of *C. glabrata*, and 100% of *C. nivariensis*. To our knowledge, this is the first study to report a synergistic effect in non-*C. albicans Candida* species. Two standardized methods were also used to assess in vitro antifungal susceptibility and two techniques of analyzing the effect of the combinations. These results align with previous findings in *C. albicans*, as Marchetti et al. [26] and Cruz et al. [25] observed synergism against 100% and 81.8% of the analyzed *C. albicans* isolates, respectively. Additional studies, including those by Liu et al. [9], Uppuluri et al. [27], and Jia et al. [13], further support the potential therapeutic efficacy of this combination. Liu et al. [9] found a synergistic effect of the FLZ-CsA combination against 66.6% of the *C. albicans* strains tested, while Uppuluri et al. [27] reported a similar synergy against the 54.5% of the studied isolates. Jia et al. [13] demonstrated that the combination also effectively disrupted biofilm formation in 24 *C. albicans* isolates, providing an enhanced treatment strategy against biofilm-associated infections. Moreover, Li et al. [28] observed synergistic effects in both fluconazole-susceptible and fluconazole-resistant *C. albicans* strains using the checkerboard assay, with particularly strong synergy in fluconazole-resistant isolates. They also reported a high level of agreement between the Loewe and Bliss models, which is consistent with our own results.

With regard to *C. glabrata*, epidemiological studies, such as those by Pfaller and Diekema [29], have reported an increase in invasive candidiasis caused by this species. This invasive candidiasis is commonly associated with high mortality in immunodeficient persons and accounting for approximately 30% of all ICU candidemia cases [30,31,32]. Most *C. glabrata* clinical isolates, while commonly exhibiting dose-dependent susceptibility (SDD) to FLZ, still show limited susceptibility to treatment [33]. Our findings support the potential therapeutic role of CsA in enhancing FLZ efficacy against *C. glabrata*, as a synergistic/additive effects against both SDD and fluconazole-resistant isolates were observed, which highlights the clinical significance of these findings.

It is worth noting that although in some cases no additive or synergistic effect was achieved in this study, the combination of CsA with FLZ was able to reduce the MIC of FLZ. This effect was observed in both *C. albicans* and non-*C. albicans Candida* species, highlighting the potential of this combination as an alternative treatment option where other antifungal therapies have failed or may be limited. On the other hand, *C. auris* was the species in which the combination had no effect compared to the administration of each of the compounds in monotherapy, nor did it contribute to reducing the MIC of FLZ, which gives an idea of the difficult clinical management of invasive infections caused by this species.

In this study we observed slight differences in the MICs when using the CLSI and EUCAST methods; these differences are generally due to methodological variations between both protocols [15,16] (glucose content, shape of the well bottom, and reading of the results at different wavelengths) with typically lower MIC values obtained with the EUCAST method compared to the CLSI method. This outcome aligns with findings reported by Cuesta et al. [34], where EUCAST yielded lower MICs, though in their study this effect was only observed at concentrations above 2 μg/mL. Notably, in our research, the reduced MIC values using the EUCAST method extended to lower concentrations, suggesting that these methodological differences may affect susceptibility assessments over a wider range of drug concentrations than previously identified.

The results obtained using the EUCAST-modified protocol for testing the combination effect showed overall agreement between the Loewe and Bliss models in most cases, aligning with findings from other researchers [28,35]. However, discrepancies in interpretation were noted for two isolates (one *C. albicans* and one *C. glabrata*), where interactions were classified as additive by the Loewe model but as synergistic by the Bliss model. This discrepancy may be explained by the greater discriminatory power of the Bliss model, as observed in the study by Lefranc et al. [36], where Bliss analysis detected synergy in the two *C. albicans* isolates previously classified as indifferent by the FICI model and also confirmed synergistic effects against two *Candida lusitaniae* isolates. Unlike the Loewe model, which depends on a fixed endpoint, the Bliss method operates independently of such points, providing an enhanced sensitivity to potential synergistic interactions.

Furthermore, our findings showed consistent synergistic effects between both checkerboard and time–kill methods for one fluconazole-susceptible (*C. albicans* NCPF 3153) and one fluconazole-resistant strain (*C. albicans* UPV 15-157). This agreement between methods supports the potential efficacy of the CsA and FLZ combination against these specific isolates, demonstrating a significant reduction in CFU counts. This result aligns with previous findings by Marchetti et al. [26], who also reported fungicidal effects of CsA-based combinations against *Candida* isolates at lower MICs than those used in our work.

Discrepancies between checkerboard and time–kill results were noted for several isolates. In the case of *C. parapsilosis* NCPF 3104, the checkerboard assay indicated a synergistic effect between CsA and FLZ, while the time–kill analysis demonstrated a reduction of only 1.78 log compared to monotherapy. Although this reduction indicates some increased activity with the combination, it is below the 2-log threshold typically required to be classified as synergistic, resulting in an indifferent interpretation by the time–kill method [21,22]. This difference may be due to the different concentration levels of CsA used in each assay, which were higher in the checkerboard method than in the time–kill experiments.

Finally, the effect of the combination was indifferent in the checkerboard assay against *C. parapsilosis* ATCC 22019 but synergistic in the time–kill analysis. Conversely, in the case of *C. albicans* UPV 15-147 the combination was synergistic in the checkerboard test but was classified as indifferent in the time–kill study. This inconsistency in the results may be attributed to the presence of clustered forms, which complicate the CFU calculations for the time–kill method, as indicated by Mukherjee et al. [37]. Additionally, when analyzing *C. albicans* ATCC 64124, the additive effect in the checkerboard results was determined to be indifferent in the time–kill curves. The observed data suggest that the combination of fluconazole (FLZ) and cyclosporine A (CsA) primarily exerts a fungistatic rather than a fungicidal effect. This observation is consistent with fluconazole’s known dose-dependent fungicidal activity, which requires higher concentrations to achieve complete eradication of fungal cells. The fungistatic behavior may reflect the inability of the tested concentrations to surpass the threshold required for fungicidal effects in certain isolates, particularly those with reduced susceptibility to fluconazole.

Previous studies have reported similar findings. For example, Li et al. [28] demonstrated that drug combinations classified as synergistic in checkerboard assays often display fungistatic rather than fungicidal activity in time–kill assays. This discrepancy was particularly evident against fluconazole-susceptible *Candida* strains. In our study, a similar trend was observed for *C. parapsilosis* ATCC 22019, where both checkerboard and time–kill assays indicated fungistatic activity.

For azole-resistant strains, such as *C. albicans* UPV 15-147 in our dataset, the fungistatic effect of the FLZ-CsA combination aligns with findings by Li et al. [28], who reported that the combination often failed to achieve fungicidal outcomes in resistant isolates. These results emphasize the importance of optimizing drug concentrations and dosing regimens to improve the fungicidal potential of this combination. In summary, the inhibition of the calcineurin pathway has been suggested as a promising target for the development of future antifungal agents. In their study, Xu et al. [38] observed that the susceptibility of *C. albicans* to high levels of extracellular calcium involves the calcineurin pathway, along with pH and homeostasis, among other factors. Blocking this pathway could enhance the antifungal efficacy of drugs targeting ergosterol biosynthesis, as there may be a correlation between these pathways. Onyewu et al. [39] demonstrated that fenpropimorph and terbinafine display synergistic activity with calcineurin inhibitors against *C. albicans*, *C. glabrata*, and *C. krusei*. However, in our study, CsA alone did not show antifungal activity, though it did significantly reduce the MIC of FLZ when used in combination. This finding highlights the potential for calcineurin pathway inhibition to improve outcomes when used in combination with other antifungal agents, as it has been demonstrated in this study where CsA has increased the effect of FLZ against some fluconazole-resistant isolates. These results make antifungal combination therapy a promising therapeutic approach and provide a starting point for investigating structural analogues of CsA that can overcome the limitation on the use of this compound, and that could be used to treat infections caused by resistant *Candida* species.

## 5. Conclusions

This study highlights the antifungal efficacy of CsA in combination with FLZ against different *Candida* species involved in invasive candidiasis for several reasons: on one hand a synergistic effect has been observed in a large number of the isolates studied, including resistant isolates belonging to seven different species; on the other hand, the combination of cyclosporine A with fluconazole reduced the MIC of fluconazole by up to 6-fold even against resistant isolates.

The usefulness of this combination of drugs could be even greater in the treatment of invasive candidiasis caused by species with common azole resistance reports, such as *C. glabrata* and *C. parapsilosis*, where synergistic and/or additive effects are observed in vitro. In addition, the fact that CsA and FLZ are well known drugs, both in their pharmacokinetics and pharmacodynamics properties and adverse effects, and that their drug–drug interactions are clearly delineated, adds value to this therapeutic combination.

Additionally, the concordance of the results obtained between the CLSI and EUCAST techniques in relation to the susceptibility data and the Loewe and Bliss methods in assessing the effect of the combination was demonstrated.

The results support the exploration of novel therapeutic strategies to address the challenges posed by *Candida* infections, particularly in resistant strains.

## Figures and Tables

**Table 1:** MIC values of fluconazole (FLZ) and cyclosporine A (CsA) alone and in combination against 37 isolates of *Candida* using the CLSI method. Interpretation determined by Loewe and Bliss model.

Isolates	MIC CLSI (μg/mL)								
	Monotherapy		In Combination		Loewe Model		Bliss Model		
	FLZ	CsA	FLZ	CsA	FICI	INT	∑SIN	∑ANT	INT
*C. albicans* ATCC 64124	>64 (R)	>128	64	2	0.52	AD	1291	−1310	AD
*C. albicans* NCPF 3153	1 (S)	>128	0.25	16	0.5	SYN	272	−154	SYN
*C. albicans* UPV 06-100	1 (S)	>128	1	0.25	1	IND	182	−167	IND
*C. albicans* UPV 06-114	1 (S)	>128	1	0.5	1	IND	244	−241	IND
*C. albicans* UPV 10-166	32 (R)	>128	2	4	0.05	SYN	954	−771	SYN
*C. albicans* UPV 10-170	>64 (R)	>128	1	2	0.01	SYN	1191	−114	SYN
*C. albicans* UPV 15-147	>64 (R)	>128	1	1	0.01	SYN	2351	−173	SYN
*C. albicans* UPV 15-154	>64 (R)	>128	1	2	0.02	SYN	2205	−487	SYN
*C. albicans* UPV 15-157	>64 (R)	>128	1	2	0.13	SYN	2336	−666	SYN
*C. albicans* UPV 15-176	>64 (R)	>128	1	>128	1.01	IND	690	−699	IND
*C. glabrata* ATCC 90030	16 (SDD)	>128	2	64	0.27	SYN	996	−728	SYN
*C. glabrata* UPV 07-185	32 (SDD)	>128	4	4	0.13	SYN	1240	−1019	SYN
*C. glabrata* UPV 07-200	32 (SDD)	>128	2	2	0.07	SYN	1589	−890	SYN
*C. glabrata* UPV 11-452	32 (R)	>128	8	64	0.13	SYN	904	−615	SYN
*C. glabrata* UPV 15-202	64 (R)	>128	4	0.25	0.04	SYN	1140	−583	SYN
*C. glabrata* UPV 16-006	8 (SDD)	>128	4	64	0.51	AD	468	−440	AD
*C. glabrata* UPV 16-032	>64 (R)	>128	4	>128	1	IND	441	−496	IND
*C. krusei* ATCC 6258	32 (R)	>128	32	0.25	0.51	AD	241	−292	AD
*C. krusei* NCPF 3321	32 (R)	>128	32	0.25	1.03	IND	247	−231	IND
*C. krusei* UPV 03-263	64 (R)	>128	64	128	1.03	IND	324	−264	IND
*C. nivariensis* CBS 9983	8 (R)	>128	2	4	0.26	SYN	1894	−1663	SYN
*C. nivariensis* CBS 9984	8 (R)	>128	2	8	0.27	SYN	1052	−747	SYN
*C. orthopsilosis* ATCC 96141	2 (S)	>128	2	0.25	1	IND	211	−191	IND
*C. orthopsilosis* UPV 09-242	2 (S)	>128	2	0.25	1.5	IND	319	−291	IND
*C. parapsilosis* ATCC 22019	1 (S)	>128	1	>128	1.5	IND	172	−189	IND
*C. parapsilosis* ATCC 90018	1 (S)	>128	0.25	>128	1.5	IND	201	−243	IND
*C. parapsilosis* ATCC MYA 4646	4 (SDD)	>128	1	32	0.31	SYN	328	−125	SYN
*C. parapsilosis* NCPF 3104	>64 (R)	>128	1	4	0.02	SYN	709	−480	SYN
*C. parapsilosis* UPV 12-241	2 (S)	>128	2	0.25	0.75	AD	166	−93	AD
*C. parapsilosis* UPV 15-177	4 (SDD)	>128	4	0.25	1.25	IND	247	−236	IND
*C. tropicalis* UPV 05-014	1 (S)	>128	1	2	1.01	IND	977	−951	IND
*C. tropicalis* UPV 09-273	>64 (R)	>128	1	2	0.02	SYN	2129	−580	SYN
*C. auris* UPV 17-213	>64 (R)	>128	>64	>128	2	IND	152	−251	IND
*C. auris* UPV 17-259	>64 (R)	>128	>64	>128	2	IND	165	−159	IND
*C. auris* UPV 17-267	>64 (R)	>128	>64	>128	2	IND	239	−268	IND
*C. auris* UPV 17-279	>64 (R)	>128	>64	>128	2	IND	301	−314	IND
*C. auris* UPV 17-281	>64 (R)	>128	>64	>128	2	IND	189	−195	IND

INT: interpretation; R: resistant; SDD: susceptible dose-dependent; S: susceptible; SYN: synergy; AD: additive; IND: indifferent.

**Table 2:** MIC values of fluconazole (FLZ) and cyclosporine A (CsA) alone and in combination against 37 isolates of *Candida* using the EUCAST method. Interpretation determined by Loewe and Bliss model.

Isolates	MIC CLSI (μg/mL)								
	Monotherapy		In Combination		Loewe Model		Bliss Model		
	FLZ	CsA	FLZ	FLZ	CsA	INT	∑SIN	∑ANT	INT
*C. albicans* ATCC 64124	>64 (R)	>128	64	4	0.52	AD	475	−485	AD
*C. albicans* NCPF 3153	1 (S)	>128	0.25	64	0.5	AD	203	−90	SYN
*C. albicans* UPV 06-100	1 (S)	>128	1	0.25	1	IND	221	−145	IND
*C. albicans* UPV 06-114	1 (S)	>128	1	0.25	1	IND	403	−415	IND
*C. albicans* UPV 10-166	32 (R)	>128	1	4	0.05	SYN	1274	−957	SYN
*C. albicans* UPV 10-170	>64 (R)	>128	1	0.25	0.01	SYN	2841	−949	SYN
*C. albicans* UPV 15-147	>64 (R)	>128	4	1	0.01	SYN	2160	−415	SYN
*C. albicans* UPV 15-154	>64 (R)	>128	2	2	0.02	SYN	2326	−620	SYN
*C. albicans* UPV 15-157	>64 (R)	>128	2	1	0.13	SYN	2409	−733	SYN
*C. albicans* UPV 15-176	>64 (R)	>128	2	>128	1.01	IND	769	−770	IND
*C. glabrata* ATCC 90030	8 (I)	>128	2	4	0.27	SYN	1096	−828	SYN
*C. glabrata* UPV 07-185	32 (I)	>128	2	2	0.13	SYN	2010	−1230	SYN
*C. glabrata* UPV 07-200	16 (I)	>128	1	2	0.07	SYN	1589	−995	SYN
*C. glabrata* UPV 11-452	64 (R)	>128	16	16	0.13	SYN	1474	−714	SYN
*C. glabrata* UPV 15-202	32 (I)	>128	1	2	0.04	SYN	2111	−1277	SYN
*C. glabrata* UPV 16-006	4 (I)	>128	2	2	0.51	AD	608	−535	SYN
*C. glabrata* UPV 16-032	>64 (R)	>128	1	>128	1	IND	2232	−2202	IND
*C. krusei* ATCC 6258	32 (R)	>128	16	4	0.51	AD	281	−359	AD
*C. krusei* NCPF 3321	64 (R)	>128	2	>128	1.03	IND	219	−195	IND
*C. krusei* UPV 03-263	64 (R)	>128	2	>128	1.03	IND	357	−299	IND
*C. nivariensis* CBS 9983	8 (R)	>128	1	2	0.13	SYN	2369	−2123	SYN
*C. nivariensis* CBS 9984	8 (R)	>128	1	4	0.14	SYN	1230	−1024	SYN
*C. orthopsilosis* ATCC 96141	1 (S)	>128	1	0.25	1	IND	507	−586	IND
*C. orthopsilosis* UPV 09-242	2 (S)	>128	1	>128	1.5	IND	875	−798	IND
*C. parapsilosis* ATCC 22019	1 (S)	>128	1	>128	1.5	IND	353	−365	IND
*C. parapsilosis* ATCC 90018	1 (S)	>128	0.25	>128	1.5	IND	190	−273	IND
*C. parapsilosis* ATCC MYA 4646	4 (I)	>128	1	16	0.31	SYN	1173	−538	SYN
*C. parapsilosis* NCPF 3104	>64 (R)	>128	2	4	0.02	SYN	921	−420	SYN
*C. parapsilosis* UPV 12-241	2 (S)	>128	1	64	0.75	AD	766	−693	AD
*C. parapsilosis* UPV 15-177	8 (R)	>128	2	>128	1.25	IND	853	−753	IND
*C. tropicalis* UPV 05-014	1 (S)	>128	1	2	1.01	IND	876	−833	IND
*C. tropicalis* UPV 09-273	>64 (R)	>128	2	2	0.02	SYN	3349	−570	SYN
*C. auris* UPV 17-213	>64 (R)	>128	>64	>128	2	IND	362	−385	IND
*C. auris* UPV 17-259	>64 (R)	>128	>64	>128	2	IND	458	−514	IND
*C. auris* UPV 17-267	>64 (R)	>128	>64	>128	2	IND	269	−287	IND
*C. auris* UPV 17-279	>64 (R)	>128	>64	>128	2	IND	502	−656	IND
*C. auris* UPV 17-281	>64 (R)	>128	>64	>128	2	IND	443	−453	IND

INT: interpretation; R: resistant; I: susceptible, increased exposure; S: susceptible; SYN: synergy; AD: additive; IND: indifferent.

## Data Availability

The original contributions presented in this study are included in the article/Supplementary Materials. Further inquiries can be directed to the corresponding author.

## Supplementary Materials

The following supporting information can be downloaded at: https://www.mdpi.com/article/10.3390/jof11020133/s1, Table S1: Clinical isolates tested in this work.
