# Supplementary material for: In Vitro Assessment of Fluconazole and Cyclosporine A Antifungal Activities: A Promising Drug Combination Against Different Candida Species
Source: J Fungi (Basel). 2025 Feb 10;11(2):133. doi: 10.3390/jof11020133 (PMC11856077; doi:10.3390/jof11020133)
Supplement: Supplementary file 1 [file jof-11-00133-s001.zip › jof-3434486-supplementary.pdf]

Supplementary material

Table S1. Clinical isolates tested in this work

Clinical isolate	Anatomical origin
Candida albicans **UPV 06-100**	Necrotizing cellulitis
Candida albicans **UPV 06-114**	Oral (prosthesis)
Candida albicans **UPV 10-166**	Clinical isolation
Candida albicans **UPV 10-170**	Clinical isolation
Candida albicans **UPV 15-147**	Vaginal
Candida albicans **UPV 15-154**	Blood culture
Candida albicans **UPV 15-157**	Oral mucose
Candida albicans **UPV 15-176**	Clinical isolation
Candida albicans **ATCC 64124**	Mouth swab
Candida albicans **NCPF 3153**	Blood culture
Candida auris **UPV 17-213**	Blood culture
Candida auris **UPV 17-259**	Blood culture
Candida auris **UPV 17-267**	Blood culture
Candida auris **UPV 17-279**	Oropharynx
Candida auris **UPV 17-281**	Urine
Candida glabrata **UPV 07-185**	Oral exudate
Candida glabrata **UPV 07-200**	Blood culture
Candida glabrata **UPV 11-452**	Sputum
Candida glabrata **UPV 15-202**	Vaginal
Candida glabrata **UPV 16-006**	Clinical isolation
Candida glabrata **UPV 16-032**	Unknown
Candida glabrata **ATCC 90030**	Blood
Candida krusei **UPV 03-263**	Blood
Candida krusei **ATCC 6258**	Sputum of patient with bronchomycosis
Candida krusei **NCPF 3321**	Unknown
Candida nivariensis **CBS 9983**	Blood culture
Candida nivariensis **CBS 9984**	Broncoalveolar lavage
Candida orthopsilosis **UPV 09-242**	Blood
Candida orthopsilosis **ATCC 96141**	Blood
Candida parapsilosis **UPV 12-241**	Vaginal
Candida parapsilosis **UPV 15-177**	Unknown
Candida parapsilosis **ATCC 22019**	Case of sprue
Candida parapsilosis **ATCC 90018**	Blood
Candida parapsilosis **ATCC MYA 4646**	Hand of healthcare worker
Candida parapsilosis **NCPF 3104**	Toe nail
Candida tropicalis **UPV 09-273**	Blood
Candida tropicalis **UPV 05-014**	Oral
